# Incidence and risk factors for the progression of proximal junctional kyphosis in degenerative lumbar scoliosis following long instrumented posterior spinal fusion

**DOI:** 10.1097/MD.0000000000004443

**Published:** 2016-08-12

**Authors:** Hui Wang, Lei Ma, Dalong Yang, Tao Wang, Sidong Yang, Yanhong Wang, Qian Wang, Feng Zhang, Wenyuan Ding

**Affiliations:** a Department of Spine Surgery, The Third Hospital of HeBei Medical University, Shijiazhuang; b XingTai People's Hospital, Xingtai; c Department of Anatomy, Basic Medical College of North China University of Science and Technology, Tangshan, China.

**Keywords:** degenerative lumbar scoliosis, obesity, osteoporosis, proximal junctional kyphosis, thoracolumbar junction, UIV

## Abstract

The aim of this study was to identify the prevalence of proximal junctional kyphosis (PJK) in degenerative lumbar scoliosis (DLS) following long instrumented posterior spinal fusion, and to search for predictable risk factors for the progression of junctional kyphosis.

In total 98 DLS patients with a minimum 2-year follow-up were reviewed prospectively. According to the occurrence of PJK at the last follow-up, patients were divided into 2 groups: PJK group and non-PJK group. To investigate risk values for the progression of PJK, 3 categorized factors were analyzed statistically: patient characteristics—preoperative data of age, sex, body mass index (BMI), bone mineral density (BMD) were investigated; surgical variables—the most proximal and distal levels of the instrumentation, the number of instrumented levels; pre- and postoperative radiographic parameters include the scoliotic angle, sagittal vertical axis, thoracic kyphosis, thoracolumbar junctional angle, lumbar lordosis, pelvic incidence, pelvic tilt, and sacral slope.

PJK was developed in 17 of 98 patients (17.3%) until to the final follow-up and were enrolled as the PJK group, and 81 patients without PJK at final follow-up were enrolled as the non-PJK group. There was no statistically significant difference between the 2 groups in age at operation (*P* = 0.900). The patient's sex was excluded in statistical analysis because of the predominance of female patients. There were statistically significant difference between the 2 groups in BMI ([25.5 ± 1.7] kg/m^2^ in the PJK group and [23.6 ± 1.9] kg/m^2^ in the non-PJK group, *P* < 0.001) and BMD ([–1.4 ± 0.8] g/cm^2^ in the PJK group and [−0.7 ± 0.3] g/cm^2^ in the non-PJK group, *P* < 0.001). No specific surgery-related variables were found to be associated with an increased risk of developing PJK, except when the most proximal instrumented vertebrae stopped at thoracolumbar junction (T11-L1). The upper instrumentation vertebrae (UIV) at thoracolumbar junction was more common in the PJK group than that in the non-PJK group (*P* = 0.007). No preoperative and early postoperative variable did reveal a statistically significant difference between the 2 groups. When included in a multivariate logistic regression model, BMI>25 kg/m^2^, osteoporosis, and UIV at thoracolumbar junction were independently associated with PJK.

In conclusion, osteoporosis, obesity, and UIV at thoracolumbar junction are risk factors for the development and progression of PJK in DLS patients following long instrumented posterior spinal fusion. Antiosteoporosis treatment extends the fusion level above the thoracolumbar region and controlling body weight before and after surgery could provide opportunities to reduce the rate of PJK and to improve therapeutic outcomes.

## 1 Introduction

Proximal junctional kyphosis (PJK) is a common complication following long instrumented spinal fusion surgery. [^1^ ^2^ ^3^] The incidence of PJK ranged from 17% to 39% in adult scoliosis patients, 27% in adolescent scoliosis patients, and 30% in Scheuermann's kyphosis patients, which depends on the study population and the duration of follow-up. [^4^ ^5^ ^6^] Varied pathogenic mechanisms may influence the development and progression of PJK; the major risk factors include older age, large preoperative sagittal parameters, use of pedicle screws, thoracoplasty procedure, greater curvature correction, posterior and anterior–posterior spinal fusion, fusion to the sacrum, low bone mineral density, and high body mass index. [^7^ ^8^ ^9^ ^10^ ^11^] The spectrum of PJK varied widely in adults from radiographic kyphosis in asymptomatic patient to spondyloptosis as a result of trauma or implant failure resulting in severe neurological deficit and back pain. ^[12]^ Kim et al ^[13]^ reported that pain was more prevalent in PJK patients with a lower improvement in the SRS pain subscore. Complete understanding of the risk factors is of great importance in minimizing the occurrence of PJK and allows surgeons to take measures for its prevention when possible.

Degenerative lumbar scoliosis (DLS) is characterized as 3-dimensional deformity, including axial rotation, coronal and sagittal vertebral tilting, and presents not only the scoliotic deformity in coronal plane, but also the lumbar hypo-lordosis in sagittal plane. DLS usually differs from other spinal scoliotic or kyphotic deformities in patient distributions and disease mechanisms, and is much more complex than general degenerative lumbar disease. Most of the patients are female, present with osteoporosis, and sometimes the long fusion level is required for scoliosis correction. Previous literature have addressed the long-term follow-up proximal junctional changes following posterior spinal instrumentation for adolescent idiopathic scoliosis (AIS), lumbar degenerative kyphosis (LDK), adult spinal deformity, and the risk factors for PJK have been determined in the respective study.[ ^1^ ^12^ ^14^] To the best of our knowledge, no studies have demonstrated long-term follow-up results of PJK following instrumented posterior spinal fusion for DLS patients. The purpose of this study is therefore to identify the prevalence of PJK after the surgical treatment of DLS, and to search for predictable risk factors for the progression of junctional kyphosis.

## 2 Materials and methods

### 2.1 Subjects

The study was approved by the Institutional Review Board of the Third Hospital of HeBei Medical University before data collection and analysis. Inclusion criteria consisted of age > 50 years at the time of surgery, degenerative scoliosis treated with instrumented segmental posterior spinal fusion at a minimum 4 motion segments, no revision operations changing level of the upper instrumentation vertebrae (UIV) and complete radiographic data. Exclusion criteria consisted of patients with spinal deformities derived from idiopathic scoliosis, ankylosing spondylitis, neuromuscular diseases, fracture, infections, or Scheuermann kyphosis. A total of 98 DLS patients with a minimum 2-year follow-up (mean 2.8 years, range 2–6) were reviewed prospectively. All operations were performed at a single institution by the senior author between September 2008 and May 2013.

### 2.2 Imaging and clinical evaluation

**Figure 1:**
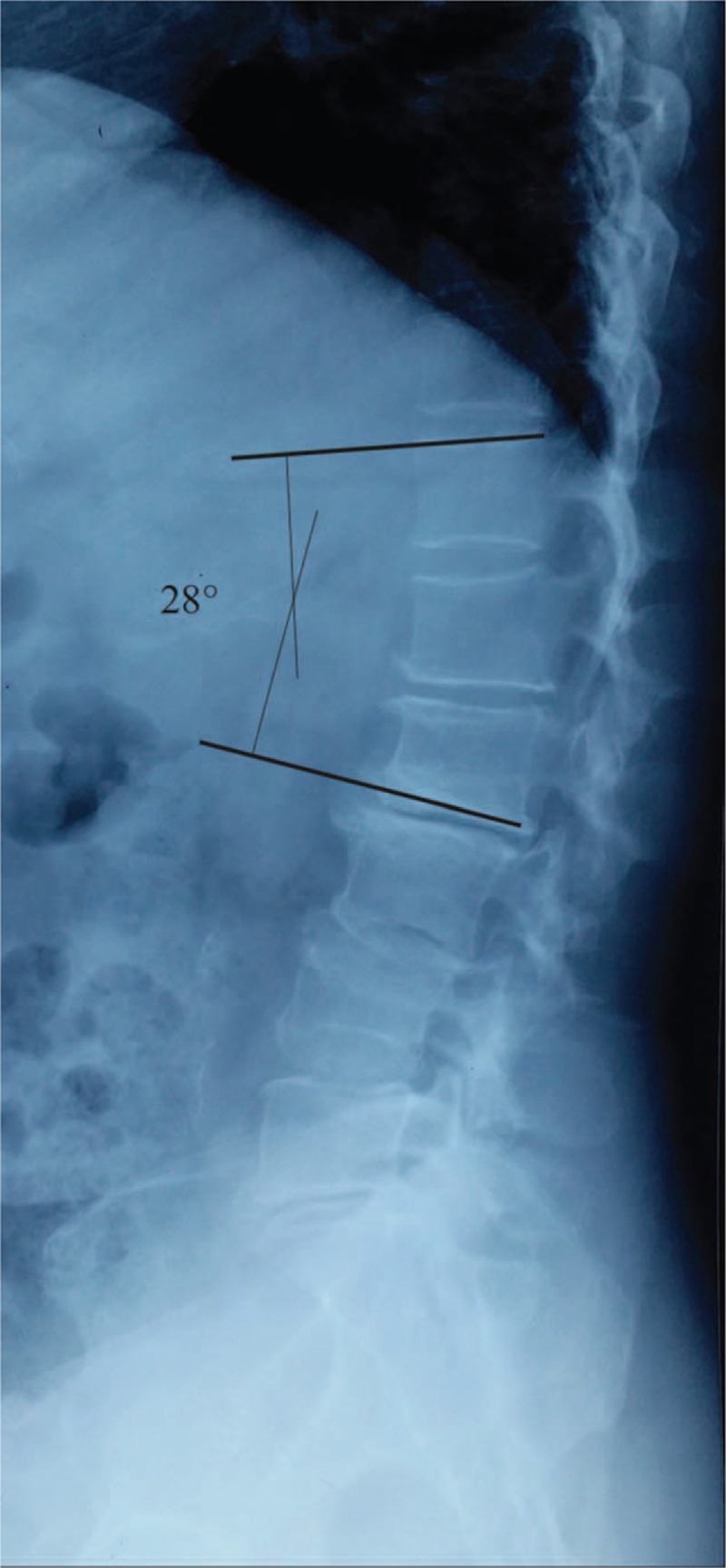
Proximal junction sagittal Cobb measurement.

The complete radiographic data include preoperation, early postoperation, 2 years postoperation, and final follow-up antero–posterior (A/P) and lateral standing 36-inch long cassette radiographs of the whole spine. Patients were asked to stand naturally with the shoulders flexed forward approximately at 60° so that their upper thoracic vertebrae could be visualized on the lateral radiograph. The proximal junctional (PJ) angle was determined as the Cobb angle between the 2 level cephalad endplates to the UIV and the caudal endplate of the UIV (Fig. 1). PJK was defined by 2 criteria: (1) the proximal junction sagittal Cobb angle >10° and (2) the proximal junction sagittal Cobb angle at least 10° greater than the preoperative measurement, the presence of both criteria was necessary to be considered abnormal. Occurrence of a spontaneous vertebral compression fracture on the proximal junctional level during the follow-up period was also regarded as PJK.

**Figure 2:**
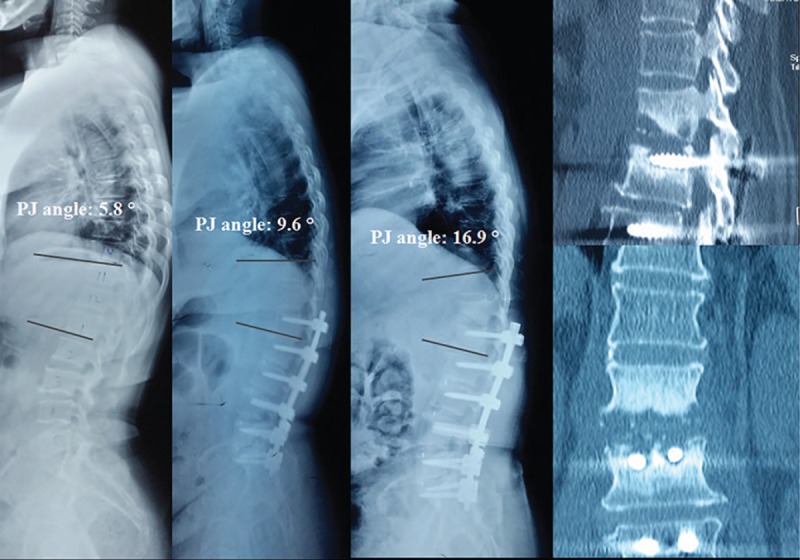
Serial radiographs with preoperative, initial postoperative, 2-year follow-up of 63-year-old female patient who had treated for DLS. The proximal junctional (PJ) angle increased from 5.8° preoperative to 9.6° in immediate postoperation, to 16.9° in 2 years follow-up. The sagittal and coronal plane CT showed UIV+1 vertebral fracture. CT = computed tomography, DLS = degenerative lumbar scoliosis, PJ = proximal junctional.

**Figure 3:**
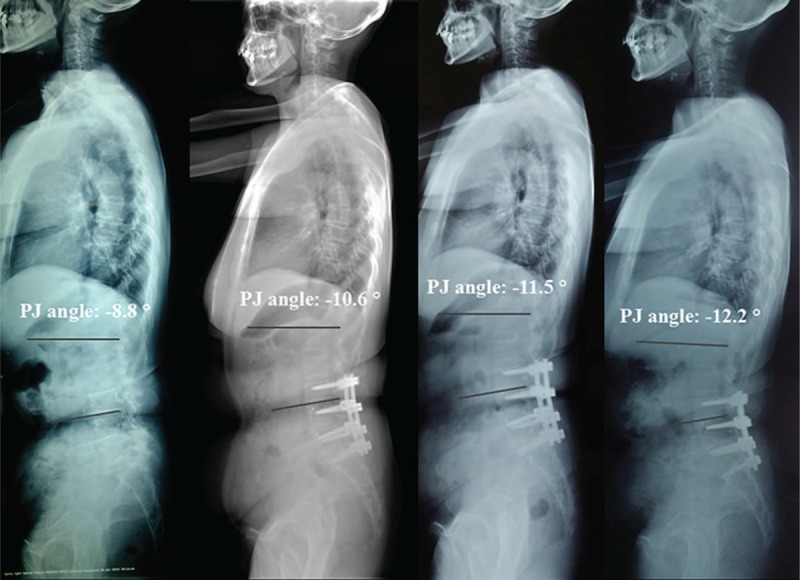
Serial radiographs with preoperative, initial postoperative, 2-year follow-up and the last follow-up of 59-year-old female patient who had treated for DLS. The PJ angle increased from –8.8° preoperative to –10.6° in immediate postoperation, to –11.5° in 2 years follow-up, and to –12.2° at final follow-up. CT = computed tomography, DLS = degenerative lumbar scoliosis.

**Figure 4:**
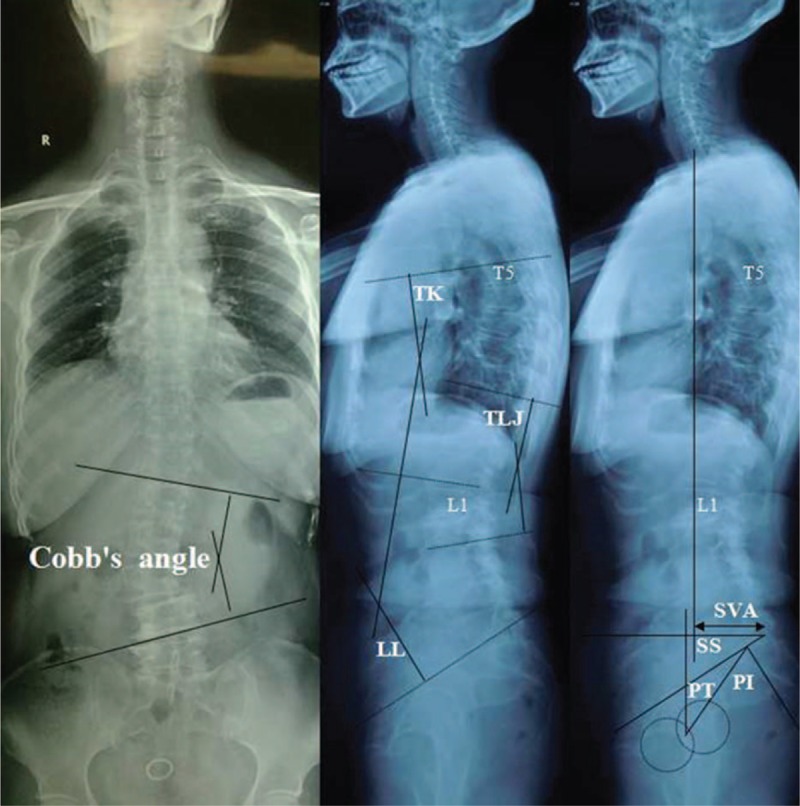
Illustration showing radiographic measurements of spinopelvic parameters included in this analysis.

According to the occurrence of PJK at the last follow-up, patients were divided into 2 groups: PJK group (Fig. 2) and non-PJK group (Fig. 3). To investigate risk values for the progression of PJK, 3 categorized factors were analyzed statistically: (1) patient characteristics—preoperative data of age, sex, body mass index (BMI), and bone mineral density (BMD). (2) Surgical variables—the most proximal and distal levels of the instrumentation, the number of instrumented levels. (3) Radiographic parameters—pre- and postoperative radiographic parameters include the scoliotic angle (SA), sagittal vertical axis (SVA), thoracic kyphosis (TK), thoracolumbar junctional (TLJ) angle, lumbar lordosis (LL), pelvic incidence (PI), pelvic tilt (PT), and sacral slope (SS) (Table 1 and Fig. 4).

**Table 1:**
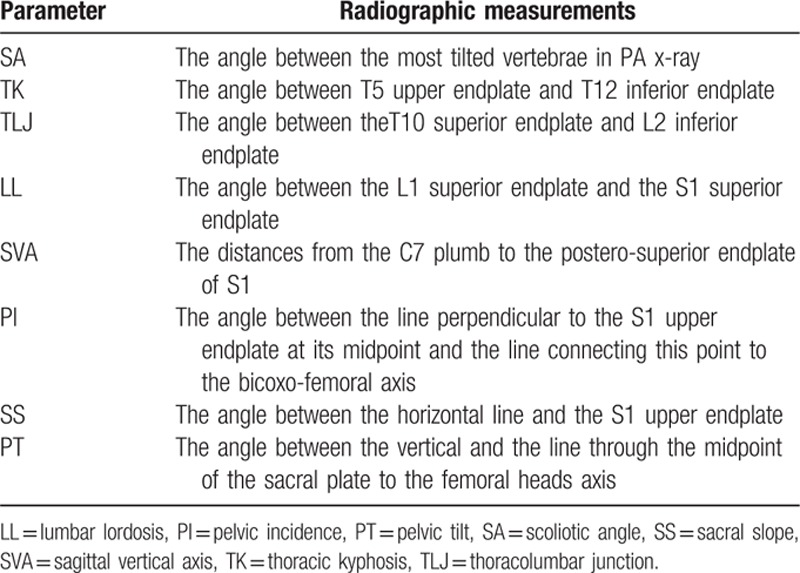
Preoperation and postoperation radiographic measurements.

### 2.3 Statistical analysis

Data were analyzed using the Statistical Product and Service Solutions software (version 13; SPSS, Chicago, IL). Continuous variables were measured as mean ± standard deviation, and categorical variables were expressed as frequency or percentages. An independent *t* test was used to analyze the difference of continuous variables between 2 groups. An χ^2^ analysis and Fisher's exact test were used to examine the differences among categorical variables. Variables with *P* values < 0.05 in the univariate analyses, as well as a number of variables selected by experts, were entered into a multivariate logistic regression model. For each variable, we computed the odds ratio (OR) with its 95% CI.

## 3 Results

PJK was developed in 17 of 98 patients (17.3%) until to the final follow-up. Eight patients experienced 10° to 14° PJ angle increase, 6 patients experienced 15° to 19° PJ angle increase, and 3 patients experienced 20° to 30° PJ angle increase. The average PJ angle increased from 3.1° preoperative to 6.8° in immediate postoperation, to 11.5° in 2 years follow-up, and to 16.2° at final follow-up in the PJK group.

There was no statistically significant difference between the 2 groups in age at operation (*P* = 0.729). The patient's sex was excluded in statistical analysis because of the predominance of female patients. The mean BMI of the PJK group was (25.5 ± 2.7) kg/m^2^, and the non-PJK group's was (23.6 ± 1.9) kg/m^2^; there was statistically significant difference between the 2 groups (*P* < 0.001). The mean BMD of the PJK group was (−1.4 ± 0.8) g/cm^2^, and the non-PJK group's was (−0.7 ± 0.3) g/cm^2^; there was also statistically significant difference between the 2 groups (*P* < 0.001). No significant differences were detected in preoperative coronal and sagittal spino-pelvic parameters between the 2 groups (Table 2).

**Table 2:**
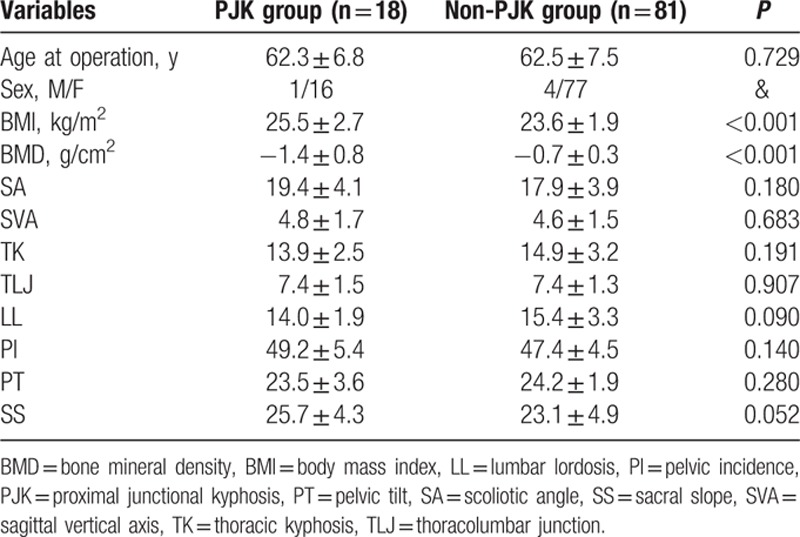
Comparison of preoperative patient characteristics between PJK and non-PJK group.

The cranial instrumented level varied from T9 to L3, and the distal level of the instrumentation was distributed from L4 to the Sacrum. Numbers of fused segments varies from 4 segments (from L1 to L5) to 9 segments (from T10 to Sacrum). No specific surgery-related variables were found to be associated with an increased risk of developing PJK, except when the most proximal instrumented vertebrae stopped at thoracolumbar junction (T11-L1). The UIV at thoracolumbar junction was more common in the PJK group than that in the non-PJK group (*P* *=* 0.007) (Table 3).

**Table 3:**
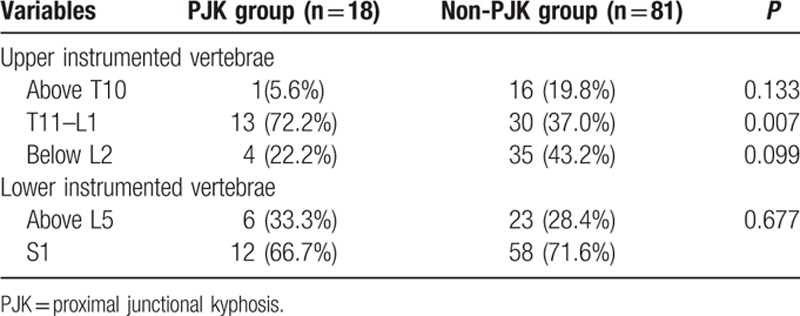
Comparison of surgical variables between PJK and non-PJK group.

The measured preoperative and early postoperative radiographic parameters related to the occurrence of PJK are summarized in Table 4. Postoperatively, both of the 2 groups got obvious SA correction and SVA reduction, with increase of TK, LL, SS, and decrease of TLJ, PT, and without change of PI. No preoperative and early postoperative variable did reveal a statistically significant difference between the 2 groups.

**Table 4:**
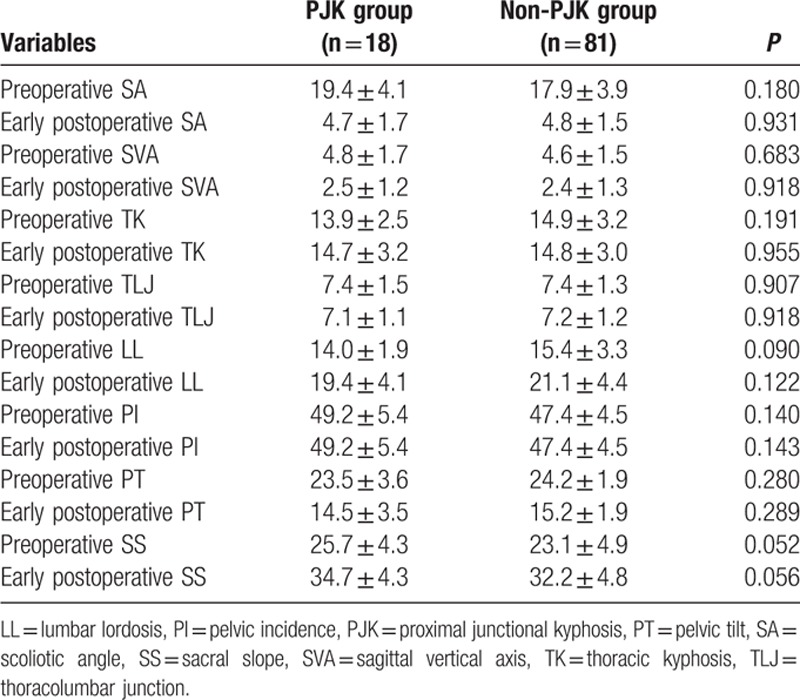
Comparison of radiographic parameters between PJK and non-PJK group.

The following variables were entered into the multivariate model: age, BMI, BMD, UIV at thoracolumbar junction, lower instrumented vertebra at sacrum, the number of instrumented levels, preoperative SA, PI, LL, and TLJ. When included in a multivariate logistic regression model, obesity, osteoporosis, and UIV at thoracolumbar junction were independently associated with PJK (Table 5).

**Table 5:**
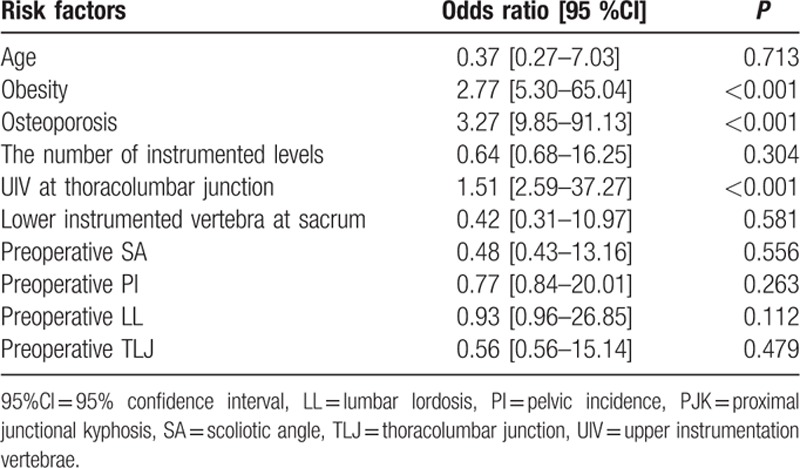
Risk factors for PJK in DLS patients, identified by multivariate analysis.

## 4 Discussion

Whether PJK is derived from the iatrogenic effect of the fusion or is a consequence of natural age-related degeneration is still difficult to state now. However, it is widely accepted that PJK is not only attributable to the passing of time and is likely multifactorial in nature, an increased understanding of the risks of PJK is pivotal to improve therapeutic outcomes. [^15^ ^16^ ^17^ ^18^] In this study, we did find that those with osteoporosis, UIV at thoracolumbar junction, BMI>25 kg/m^2^ should be monitored closely for the development and progression of PJK in DLS patients.

Osteoporosis is a chronic disease generally associated with aging and affects a large amount of persons, especially the elders. It is characterized by the reduction of the bone mass and the modification of the bone architecture, and increases the risk of vertebral fracture. In this study, we find the BMD was lower in the PJK group than that in the non-PJK group and the UIV+1 vertebral fracture was common in PJK patients. Although PJK occurs in both the adult and pediatric patients, it represents a distinct phenomenon in the 2 populations. In children with adolescent idiopathic scoliosis undergoing spinal fusion surgery, PJK often manifests itself as a kyphotic change in the disc space above the fusion. In addition to the disc change, PJK in adults also presents fractures and subluxations above the fusion, and this can lead to proximal junction kyphosis or failure causing pain, deformity, and instability. [^19^ ^20^ ^21^ ^22^] It is still a controversial whether vertebral cement augmentation is an effective method to prevent PJK following long segment spinal fusion. Hart et al found that 15.3% of the nonaugmented patients demonstrated PJK secondary to vertebrae collapse, whereas cement augmented patients did not present PJK, suggesting that cement augmentation was helpful in preventing or reducing the risk of PJK. ^[23]^ However, there are 2 drawbacks to cement augmentation. First, it reduces nutrient supply to intervertebral disc and accelerates degenerative disc disease. Second, cement can alter load transfer, provoke fractures in adjacent vertebrae, and facilitate collapse of adjacent vertebrae. Kayanja et al ^[24]^ compared the biomechanics of augmenting different numbers of vertebral levels and found that stiffness and strength of the construct were dependent mainly on bone mineral density instead of the number of vertebral levels augmented. Therefore, we believe that the valuable method for preventing PJK in DLS patients is antiosteoporosis treatment, instead of the cement augmentation. The methods for increasing bone density includes calcium/vitamin D supplementation, drugs of the bisphosphonate family that prevent bone resorption, all the methods need to be implemented both in preoperative and postoperative periods. Preoperative goal is to increase the bone density as much as possible, severe osteoporosis patients should not be excluded from the benefit of surgery, less invasive procedure (discectomy or laminectomy) instead of long instrumented posterior spinal fusion should be considered.

In our study, all patients are instrumented posteriorly, and we find that improper selection of the UIV is closely related to the occurrence of PJK. Decision making on the proximal fusion level was based in part on the status of the vertebra, the magnitude of the coronal curve, suprajacent disc space, shoulder balance, and apical deviation of the curve with coronal imbalance and regional sagittal condition. Choosing the neutral and stable vertebra as the UIV can reduce revision prevalence in adult deformity patients. ^[19]^ We preferred to stop at the stable, neutral, horizontal vertebra with a stable suprajacent disc in the coronal plane and to avoid any vertebra with compression fracture, or angular suprajacent disc in the sagittal plane. However, when stopping instrumentation around thoracolumbar junction, we find the incidence of PJK is quite high, and it can be explained by 2 possible reasons. On the one hand, the potential hazard of the concentration stress due to anatomical characteristics. Thoracolumbar junction is a transitional area from kyphosis to lordosis, which has a vulnerability from transitional force. Facet joints of thoracic spine are more coronally oriented than lumbar spine, and the rib cage can restrict spinal motion end at thoracolumbar junction. The complex biomechanics at the thoracolumbar junction with the transition from immobile to mobile motion segments certainly may play a role in degeneration of the proximal junction. On the other hand, the concentration stress following the use of internal fixation, especially the long segment instrumentation. Both animal and cadaveric studies detected the increased motion and mechanical stress adjacent to fusion constructs, which may increase the risk of PJK. ^[25]^ Dynamic stabilization with less stiff transition rod at the top to reduce rigidity of the construct proximally may be of some value, but appropriate attention should be paid to the increased likelihood of pseudarthrosis because of the increased junctional micro-motion due to the reduced proximal construct stiffness. ^[26]^ Our previous study have found that the changes in spinopelvic parameters and pelvic compensatory mechanisms differ according to PI in patients with DLS, restoration of LL based on individual PI could help in accomplishing a balanced spinopelvic alignment. Instrumentation within the lumbar region to realign the lumbar lordosis is enough to relieve the pelvic compensation in low PI, long-segment instrumentation involving the thoracolumbar region (the upper instrumented level in T10 or above) is necessary in high PI. ^[27]^ Therefore, the PI value should be considered in surgical planning for DLS patients, and location of the most proximal instrumented vertebra should be individual.

BMI is an objective and simple indicator, and values > 25 and 30 kg/m^2^ define overweight and obesity, respectively. A study by Liuke et al ^[28]^ has provided evidence that BMI > 25 kg/m^2^ increases the risk of lumbar disk degeneration. Ou et al ^[29]^ also found that BMI is a risk factor for adjacent segment disease in patients undergoing lumbar fusion for degenerative spine diseases. Our data suggest that patients with BMI > 25 kg/m^2^ undertake enormous risk in the development and progression of PJK. On the one hand, increased loading of the spine causes the intervertebral disks to lose height and ability to absorb a force, resulting in abnormal loading on surrounding facet joints and spinal ligaments. If obesity imposes a burden on the vertebral column for a long time, it leads to systemic inflammatory changes by the release of adipocytokines affecting the musculoskeletal system that may initiate degenerative changes in the vertebral column. ^[30]^ On the other hand, the paraspinal muscle strength of obesity patients is weak when compared to the healthy adults. It is a necessity to strip the muscles from the spinous process and laminae in the surgery procedure; postoperative adhesion may decrease the muscle function. If the muscles cannot afford enough strength to maintain upright posture, it may accelerate the degeneration of disc and articular process, especially in the segment above the fusion level. With these changes in intradiskal pressure, facet contact loading and paraspinal muscle fatigue, obesity potentially adds more stress to the adjacent levels above the fusion and accelerates the degenerative process of disc above the UIV. Obesity may not only be a risk factor related to the natural degeneration of spine, but also may play an important role in the occurrence of PJK. Therefore, controlling body weight before and after surgery could provide opportunities to reduce the rate of PJK and to improve therapeutic outcomes. Obesity patients should not be excluded from the benefit of surgery as well, and preoperative goal is trying to decrease the BMI value to be within the normal range, the important thing they need to do is to increase the paraspinal muscle function all through the perioperative period.

There were several potential limitations in this study. First, only Chinese Han individuals were included in this study and Ethnic variation was not covered. Second, the number of patients was relatively small, and the study may be underpowered to detect the significance of some risk factors. Even with these issues in this study, we find that those with osteoporosis, obesity, UIV at thoracolumbar junction are risk factors for the development and progression of PJK in DLS patients following long instrumented posterior spinal fusion. Anti-osteoporosis treatment, extend the fusion level above the thoracolumbar region and controlling body weight before and after surgery could provide opportunities to reduce the rate of PJK and to improve therapeutic outcomes. Our data is of great value in decision making and surgical planning for both spinal surgeon and DLS patients.

## References

[1] Yagi M King AB Boachie-Adjei O Incidence, risk factors and natural course of proximal junctional kyphosis: surgical outcomes review of adult idiopathic scoliosis. Minimum 5 years follow-up. *Spine (Phila Pa 1976)* 2012; 37:1479–1489. 22357097 10.1097/BRS.0b013e31824e4888

[2] Kim HJ Lenke LG Shaffrey CI Proximal junctional kyphosis as a distinct form of adjacent segment pathology after spinal deformity surgery: a systematic review. *Spine (Phila Pa 1976)* 2012; 37:S144–S164. 22885829 10.1097/BRS.0b013e31826d611b

[3] Mendoza-Lattes S Ries Z Gao Y Proximal junctional kyphosis in adult reconstructive spine surgery results from incomplete restoration of the lumbar lordosis relative to the magnitude of the thoracic kyphosis. *Iowa Orthop J* 2011; 31:199–206. 22096442 PMC3215136

[4] Wang J Zhao Y Shen B Risk factor analysis of proximal junctional kyphosis after posterior fusion in patients with idiopathic scoliosis. *Injury* 2010; 41:415–420. 20106476 10.1016/j.injury.2010.01.001

[5] Denis F Sun EC Winter RB Incidence and risk factors for proximal and distal junctional kyphosis following surgical treatment for Scheuermann kyphosis: minimum five-year follow-up. *Spine (Phila Pa 1976)* 2009; 34:E729–E734. 19752692 10.1097/BRS.0b013e3181ae2ab2

[6] Kim HJ Bridwell KH Lenke LG Patients with proximal junctional kyphosis requiring revision surgery have higher postop lumbar lordosis and larger sagittal balance corrections. *Spine (Phila Pa 1976)* 2014; 39:E576–E580. 24480958 10.1097/BRS.0000000000000246

[7] Bridwell KH Lenke LG Cho SK Proximal junctional kyphosis in primary adult deformity surgery: evaluation of 20 degrees as a critical angle. *Neurosurgery* 2013; 72:899–906. 23407291 10.1227/NEU.0b013e31828bacd8

[8] Yagi M Akilah KB Boachie-Adjei O Incidence, risk factors and classification of proximal junctional kyphosis: surgical outcomes review of adult idiopathic scoliosis. *Spine (Phila Pa 1976)* 2011; 36:E60–E68. 21192216 10.1097/BRS.0b013e3181eeaee2

[9] Maruo K Ha Y Inoue S Predictive factors for proximal junctional kyphosis in long fusions to the sacrum in adult spinal deformity. *Spine (Phila Pa 1976)* 2013; 38:E1469–E1476. 23921319 10.1097/BRS.0b013e3182a51d43

[10] Cammarata M Aubin CE Wang X Biomechanical risk factors for proximal junctional kyphosis: a detailed numerical analysis of surgical instrumentation variables. *Spine (Phila Pa 1976)* 2014; 39:E500–E507. 24480964 10.1097/BRS.0000000000000222

[11] O'Shaughnessy BA Bridwell KH Lenke LG Does a longfusion “T3-sacrum” portend a worse outcome than a short fusion “T10-sacrum” in primary surgery for adult scoliosis? *Spine (Phila Pa 1976)* 2012; 37:884–890. 21971131 10.1097/BRS.0b013e3182376414

[12] Samuel K Cho John I Shin Yongjung F Kim Proximal junctional kyphosis following adult spinal deformity surgery. *Eur Spine J* 2014; 23:2726–2736. 25186826 10.1007/s00586-014-3531-4

[13] Kim HJ Bridwell KH Lenke LG Proximal junctional kyphosis results in inferior SRS pain subscores in adult deformity patients. *Spine (Phila Pa 1976)* 2013; 38:896–901. 23232215 10.1097/BRS.0b013e3182815b42

[14] Lee JH Kim JU Jang JS Analysis of the incidence and risk factors for the progression of proximal junctional kyphosis following surgical treatment for lumbar degenerative kyphosis: minimum 2-year follow-up. *Brit J Neurosurg* 2014; 28:252–258. 24313308 10.3109/02688697.2013.835369

[15] Lee JC Kim Y Soh JW Risk factors of adjacent segment disease requiring surgery after lumbar spinal fusion: comparison of posterior lumbar interbody fusion and posterolateral fusion. *Spine (Phila Pa 1976)* 2014; 39:E339–E345. 24365899 10.1097/BRS.0000000000000164

[16] Hikata T Kamata M Furukawa M Risk factors for adjacent segment disease after posterior lumbar interbody fusion and efficacy of simultaneous decompression surgery for symptomatic adjacent segment disease. *J Spinal Disord Tech* 2014; 27:70–75. 22460400 10.1097/BSD.0b013e31824e5292

[17] Hostin R McCarthy I O’Brien M Incidence, mode, and location of acute proximal junctional failures following surgical treatment for adult spinal deformity. *Spine (Phila Pa 1976)* 2013; 38:1008–1015. 22986834 10.1097/BRS.0b013e318271319c

[18] Maruo K Ha Y Inoue S Predictive factors for proximal junctional kyphosis in long fusions to the sacrum in adult spinal deformity. *Spine (Phila Pa 1976)* 2014; 38:E1469–E1476. 23921319 10.1097/BRS.0b013e3182a51d43

[19] Kim YJ Bridwell KH Lenke LG Is the T9, T11, or L1 the more reliable proximal level after adult lumbar or lumbosacral instrumented fusion to L5 or S1? *Spine (Phila Pa 1976)* 2007; 32:2653–2661. 18007240 10.1097/BRS.0b013e31815a5a9d

[20] Bastian L Lange U Knop C Evaluation of the mobility of adjacent segments after posterior thoracolumbar fixation: a biomechanical study. *Eur Spine J* 2001; 10:295–300. 11563614 10.1007/s005860100278 PMC3611504

[21] Cahill PJ Wang W Asghar J The use of a transition rod may prevent proximal junctional kyphosis in the thoracic spine after scoliosis surgery: a finite element analysis. *Spine (Phila Pa 1976)* 2012; 37:E687–E695. 22210013 10.1097/BRS.0b013e318246d4f2

[22] Cammarata M Aubin CÉ Wang X Biomechanical risk factors for proximal junctional kyphosis: a detailed numerical analysis of surgical instrumentation variables. *Spine (Phila Pa 1976)* 2014; 39:E500–E507. 24480964 10.1097/BRS.0000000000000222

[23] Hart RA Prendergast MA Roberts WG Proximal junctional acute collapse cranial to multi-level lumbar fusion: a cost analysis of prophylactic vertebral augmentation. *Spine J* 2008; 8:875–881. 18375188 10.1016/j.spinee.2008.01.015

[24] Kayanja MM Schlenk R Togawa D The biomechanics of 1, 2, and 3 levels of vertebral augmentation with polymethylmethacrylate in multilevel spinal segments. *Spine (Phila Pa 1976)* 2006; 31:769–774. 16582850 10.1097/01.brs.0000207466.40955.31

[25] Bastian L Lange U Knop C Evaluation of the mobility of adjacent segments after posterior thoracolumbar fixation: a biomechanical study. *Eur Spine J* 2001; 10:295–300. 11563614 10.1007/s005860100278 PMC3611504

[26] Cahill PJ Wang W Asghar J The use of a transition rod may prevent proximal junctional kyphosis in the thoracic spine after scoliosis surgery: a finite element analysis. *Spine (Phila Pa 1976)* 2012; 37:E687–E695. 22210013 10.1097/BRS.0b013e318246d4f2

[27] Hui Wang Lei Ma Da-Long Yang Radiological analysis of degenerative lumbar scoliosis in relation to pelvic incidence. *Int J Clin Exp Med* 2015; 8:22345–22351. 26885212 PMC4729998

[28] Liuke M Solovieva S Lamminen A Disc degeneration of the lumbar spine in relation to overweight. *Int J Obes (Lond)* 2005; 29:903–908. 15917859 10.1038/sj.ijo.0802974

[29] Ou CY Lee TC Lee TH Impact of body mass index on adjacent segment disease after lumbar fusion for degenerative spine disease. *Neurosurgery* 2015; 76:396–401. 25603108 10.1227/NEU.0000000000000627

[30] Shiri R Solovieva S Husgafvel-Pursiainen K The role of obesity and physical activity in non-specific and radiating low back pain: the Young Finns study. *Semin Arthritis Rheum* 2013; 42:640–650. 23270761 10.1016/j.semarthrit.2012.09.002

